# Disseminated melioidosis in early pregnancy - an unproven cause of foetal loss

**DOI:** 10.1186/s12879-020-4937-8

**Published:** 2020-03-06

**Authors:** Chee Yik Chang, Nina Lee Jing Lau, Bart J. Currie, Yuwana Podin

**Affiliations:** 1 Medical Department, Kapit Hospital, Ministry of Health, Kapit, Malaysia; 2 Obstetrics and Gynaecology Department, Kapit Hospital, Ministry of Health, Kapit, Malaysia; 3 Global and Tropical Health Division, Menzies School of Health Research, Charles Darwin University, Darwin, Australia; 4 Institute of Health and Community Medicine, University Malaysia Sarawak, Kota Samarahan, Sarawak Malaysia

**Keywords:** Melioidosis, *Burkholderia pseudomallei*, Pregnancy, Abortion

## Abstract

**Background:**

Melioidosis is a potentially life-threatening infection caused by the Gram-negative bacterium *Burkholderia pseudomallei*. Melioidosis is difficult to diagnose due to its diverse clinical manifestations, which often delays administration of appropriate antibiotic therapy.

**Case presentation:**

Melioidosis is uncommon in pregnancy but both spontaneous abortion and neonatal melioidosis have been reported. We report a case of bacteraemic melioidosis in a young woman with a subsequent spontaneous abortion, with *B. pseudomallei* cultured from a high vaginal swab as well as blood.

**Conclusion:**

It remains unclear in this and previously reported cases as to whether the maternal melioidosis was sexually transmitted.

## Background

Melioidosis is an infection caused by the Gram-negative bacterium, *Burkholderia pseudomallei*. It is an endemic disease in northern Australia and Southeast Asian countries, especially Thailand and Malaysia, and is being increasingly recognised in other regions globally [1].

Melioidosis can virtually affect any organ in the body and can present with diverse clinical manifestations, including pneumonia, genitourinary infection, skin and soft tissue infection, internal organ abscesses, neurological melioidosis, septic arthritis, and fulminant sepsis without evident focus [2]. Despite improvements in antibiotic therapy, melioidosis is still associated with a high mortality attributable to severe sepsis and its complications [3].

Among the well-recognized modes of acquisition are inhalation, ingestion, and inoculation, but the relative contributions of each are yet to be determined [3]. Person-to-person transmission of *B. pseudomallei* is very uncommon. Sexual transmission in particular, has been suggested but has not been definitively established as a mode of infection [3, 4]. There was one reported case of possible sexual transmission from a returned United States serviceman to his partner. He was diagnosed with culture-confirmed *B. pseudomallei* prostatitis. Upon further investigations, both the patient and his wife demonstrated high indirect haemagglutination assay (IHA), which was considered suggestive of recent *B. pseudomallei* infection [5]. Nevertheless the wife was asymptomatic and her vaginal and cervical cultures were negative for *B. pseudomallei.* Therefore, this case report which is often quoted as evidence of sexual transmission of *B. pseudomallei* remains speculative and unconfirmed.

There has been very little clinical evidence on pregnancy outcomes in women with melioidosis, and it is unknown if pregnant women are specifically more susceptible to *B. pseudomallei* or if they experience more severe disease [6]. We present a case of bacteraemic melioidosis with splenic abscesses in a young, healthy woman complicated with early pregnancy loss, with *B. pseudomallei* recovered from the blood and high vaginal swab (HVS) cultures.

## Case presentation

A 25-year-old housewife, with no prior medical illness presented to Kapit Hospital, Sarawak, Malaysian Borneo, with fever associated with dysuria, urinary frequency and vaginal discharge 1 week prior. She denied cough, shortness of breath or chest pain. She divorced 4 years prior, and was living with a healthy male partner and her two children.

Upon arrival at the hospital, her general condition was stable. Physical examination revealed a blood pressure of 132/76 mmHg, pulse rate of 123 beats/min and temperature of 38.6 °C. Her respiratory rate was 20 breaths/min and the oxygen saturation on room air measured by pulse oximetry was 96%. The remainder of the physical examination was unremarkable.

Haematological analysis showed a haemoglobin of 13 g/dL, white blood cell count of 19 × 10^3^/μL and platelet count of 431 × 10^3^/μL. She was noted to have mild renal impairment with a raised serum creatinine at 129 μmol/L, while the electrolytes and liver function tests were within the normal range. The blood gas analysis revealed a compensated metabolic acidosis with good arterial oxygenation (pH 7.41, PaO_2_ 107 mmHg, PaCO_2_ 21 mmHg, HCO_3_ 13 mmol/L, base excess - 12 mmol/L). Urinalysis revealed leukocyturia and the presence of *Trichomonas vaginalis* identified by microscopic examination. A urine pregnancy test was performed, and the result was positive. Serological tests for leptospirosis and dengue were negative and so were the hepatitis B, C and HIV tests. The chest radiograph was normal.

She was subsequently admitted to the medical ward for further management. She was started on first-line antibiotics, intravenous amoxicillin-clavulanic acid and metronidazole as treatment for a presumed urinary tract infection and trichomoniasis respectively. However, her condition did not improve after 3 days of treatment as persistent fever and a worsening trend of metabolic acidosis were observed (pH 7.36, HCO_3_ 8 mmHg, base excess - 17 mmol/L). An abdominal ultrasonography revealed multiple splenic abscesses and normal pelvic organs. Her antibiotics were changed to intravenous ceftazidime 2 g 8-hourly as empirical treatment for melioidosis.

A detailed gynaecological examination was performed, followed by sampling of HVS for culture. Transvaginal ultrasound scan demonstrated an intrauterine pregnancy of 6 weeks and 4 days period of gestation but of unknown viability. Serial serum beta-human chorionic gonadotropin (β-hCG) was employed to determine its viability. The β-hCG test taken at 0-h and 48-h showed a reducing trend, which was indicative of a failing pregnancy. Two weeks later, she had an uneventful complete spontaneous abortion. Culture and histopathological examination of the products of conception did not reveal any significant pathology and there was no evidence of foetal infection with *B. pseudomallei.*

Blood and HVS cultures yielded *B. pseudomallei*, grown on modified Francis culture medium without gentamicin, as the *B. pseudomallei* isolates from Sarawak are predominantly susceptible to aminoglycosides [7]. Preliminary identification using API2ONE test (Biomerieux) and Gram staining identified *B. pseudomallei,* which was later confirmed by real-time PCR targeting the type III secretion system [8]. The organism was confirmed to be susceptible to ceftazidime, amoxicillin-clavulanic acid and trimethoprim-sulfamethoxazole by the Etest (Biomerieux, France) according to CLSI standard. ELISA for the detection of IgM against *B. pseudomallei* was negative. The urine culture was negative. She responded well to treatment, with fever and metabolic acidosis resolving by day 10. She received intravenous ceftazidime for a total of 4 weeks and repeat abdominal ultrasonography upon completion of intensive therapy showed resolution of the splenic abscesses. She then completed a 20-week course of oral doxycycline and trimethoprim-sulfamethoxazole as eradication phase treatment according to the local treatment protocol [9]. During follow-up review, she remained well and showed no signs of disease recurrence.

The patient’s partner was totally healthy and asymptomatic. He denied having fever, penile discharge, genital ulcer or dysuria. He was offered screening for sexually transmitted diseases and melioidosis, but declined. Hence, we were unable to establish the potential sexual transmission route of *B. pseudomallei* in this case.

## Discussion and conclusion

Melioidosis is caused by *B. pseudomallei*, which is a facultative intracellular Gram-negative, saprophytic bacterium, commonly found in soil or contaminated water [10]. Melioidosis is associated with a high mortality even in a developed country like Australia, with a mortality rate of approaching 20% [11]. Since the clinical manifestations of melioidosis are not distinctive and may range from acute septicaemia to a chronic suppurative disease, a high index of suspicion is required for the diagnosis of melioidosis. In our patient, there was a raised clinical suspicion for melioidosis as she presented with sepsis and splenic abscesses, which did not respond to first-line antibiotics. Early initiation of ceftazidime in this case proved to be an important contributory factor to the favourable survival outcome.

Bacteraemic melioidosis carries a poor prognosis. In earlier data from the Darwin study, melioidosis caused bacteraemia in almost half of all melioidosis cases with a mortality rate of 37%, compared with a mortality of 4% for patients with non-bacteraemic melioidosis [11]. Risk factors for melioidosis include diabetes mellitus, hazardous alcohol use, preexisting renal disease, immunosuppressive therapy including steroids and thalassaemia [10–13]. Occupational or recreational exposure to contaminated soil or water is the usual infecting event [10, 12]. However, patients without risk factors can also be infected with melioidosis. A review of local data in Malaysia revealed an absence of risk factor in 15–42% of cases diagnosed with melioidosis [13]. Our patient was a young, immunocompetent individual with no apparent risk factors for melioidosis. Therefore, absence of risk factors does not exclude melioidosis infection and this should not delay the commencement of appropriate empirical antibiotics in patients with suspected melioidosis.

A positive growth of *B. pseudomallei* in HVS cultures is an unusual finding and a key in understanding the possible pathogenesis in this case. Our patient presented with features of urinary tract infection and sexually transmitted disease (trichomoniasis). This means that *B. pseudomallei* could have been acquired from her partner through sexual transmission, with subsequent bacteraemic spread and seeding of the spleen with development of splenic abscesses. Her partner who was asymptomatic at the time of encounter might have been colonized by *B. pseudomallei*. Another possible explanation for her melioidosis would be haematogenous spread from her otherwise-acquired bacteraemic infection to the genitourinary tract as well as the spleen, and thus presence of *B. pseudomallei* in vaginal secretions. *B. pseudomallei* can be isolated from rectal swabs during acute human infection with melioidosis but occurred rarely in asymptomatic individuals [14]. Meanwhile, vaginal carriage of *B. pseudomallei* has not been reported previously, hence the presence of *B. pseudomallei* in the vagina is of uncertain relevance in this present case.

Pregnancy is not a known predisposing factor for melioidosis [15]. Three previously reported cases in pregnancy include perinatal transmission at 32 weeks from a mother whose melioidosis was diagnosed postpartum, a case of fatal bronchopneumonia with spontaneous abortion at 16 weeks gestation, and a case of placental infection leading to preterm delivery in a mother diagnosed with melioidosis septicaemia and pneumonia in pregnancy [15–17]. Webling reported on a 32-year-old lady at 24 weeks of pregnancy who presented with cystitis, vaginal discharge and an indolent ulcer of the thigh. *B. pseudomallei* was isolated from her urine and vaginal discharge. She absconded after a short course of treatment and had an abortion a few weeks later [4]. A systematic review identified 22 published cases of neonatal melioidosis, with pneumonia, bacteraemic infection and meningitis being common. Identified routes of infection include vertical transmission, breastfeeding, community-acquired postpartum exposure, and healthcare-associated exposure. Neonatal melioidosis is associated with an extremely high overall mortality [18].

These documented cases showed that infection with *B. pseudomallei* might lead to adverse pregnancy outcomes. *B. pseudomallei* infection in pregnancy can cause intrauterine infection and subsequently foetal death and abortion, and transplacental melioidosis has also been described in animals [19, 20]. However, in our patient, culture and histopathological examination of the products of conception did not reveal any significant pathology. We presume that the severity of the infection with bacteraemia and ongoing metabolic acidosis were significant contributory factors to the poor pregnancy outcome in this case, rather than specific infection of the foetus with *B. pseudomallei* [21].

Management of melioidosis in a resource-limited setting is extremely challenging and clinicians working in endemic areas must maintain a high index of suspicion for melioidosis in order to reduce the disease-related morbidity and mortality. *B. pseudomallei* detected in a high vaginal swab as in the case reported here, might be the source of systemic infection or might simply result from haematogenous spread. This case has therefore not confirmed that sexual transmission of *B. pseudomallei* occurs*.* Irrespective of how it is acquired during pregnancy, melioidosis poses a higher risk of adverse pregnancy outcomes which includes miscarriage.

## Data Availability

Not applicable.

## Abbreviations

HVS: High vaginal swab
IHA: Indirect haemagglutination assay
β-hCG: beta-human chorionic gonadotropin
